# Synthesis and Evaluation of ^18^F-Labeled Peptide for Gonadotropin-Releasing Hormone Receptor Imaging

**DOI:** 10.1155/2019/5635269

**Published:** 2019-03-07

**Authors:** Shun Huang, Hongsheng Li, Yanjiang Han, Lilan Fu, Yunyan Ren, Yin Zhang, Youcai Li, Penghui Sun, Meng Wang, Hubing Wu, Quanshi Wang, Kongzhen Hu

**Affiliations:** Nanfang PET Center, Nanfang Hospital, Southern Medical University, 1838 Guangzhou Avenue North, Guangzhou, Guangdong 510515, China

## Abstract

The gonadotropin-releasing hormone (GnRH) receptor is overexpressed in the majority of tumors of the human reproductive system. The purpose of this study was to develop an ^18^F-labeled peptide for tumor GnRH receptor imaging. In this study, the GnRH (pGlu^1^-His^2^-Trp^3^-Ser^4^-Tyr^5^-Gly^6^-Leu^7^-Arg^8^-Pro^9^-Gly^10^-NH_2_) peptide analogues FP-d-Lys^6^-GnRH (FP = 2-fluoropropanoyl) and NOTA-P-d-Lys^6^-GnRH (*P* = ethylene glycol) were designed and synthesized. The IC_50_ values of FP-d-Lys^6^-GnRH and NOTA-P-d-Lys^6^-GnRH were 2.0 nM and 56.2 nM, respectively. 4-Nitrophenyl-2-[^18^F]fluoropropionate was conjugated to the *ε*-amino group of the d-lysine side chain of d-Lys^6^-GnRH to yield the new tracer [^18^F]FP-d-Lys^6^-GnRH with a decay-corrected yield of 8 ± 3% and a specific activity of 20−100 GBq/*µ*mol (*n*=6). Cell uptake studies of [^18^F]FP-d-Lys^6^-GnRH in GnRH receptor-positive PC-3 cells and GnRH receptor-negative CHO-K1 cells indicated receptor-specific accumulation. Biodistribution and PET studies in nude mice bearing PC-3 xenografted tumors showed that [^18^F]FP-d-Lys^6^-GnRH was localized in tumors with a higher uptake than in surrounding muscle and heart tissues. Furthermore, the metabolic stability of [^18^F]FP-d-Lys^6^-GnRH was determined in mouse blood and PC-3 tumor homogenates at 1 h after tracer injection. The presented results indicated a potential of the novel tracer [^18^F]FP-d-Lys^6^-GnRH for tumor GnRH receptor imaging.

## 1. Introduction

Gonadotropin-releasing hormone (GnRH), also known as luteinizing hormone-releasing hormone (LHRH), is a hormonal decapeptide (*p*Glu-His-Trp-Ser-Tyr-Gly-Leu-Arg-Pro-Gly-NH_2_) produced by the hypothalamus [1]. It plays an important role in the control of mammalian reproduction. Furthermore, GnRH may have a role as a modulator of cell growth and metastasis in a number of human malignant tumors, including cancers of the breast, ovary, endometrium, and prostate [2]. GnRH activity is mediated by a specific transmembrane GnRH receptor that belongs to the G-protein-coupled receptor (GPCR) family [3–5]. The GnRH receptor is overexpressed in malignant tumors, including cancers of the breast, ovary, endometrium, prostate, larynx, melanomas, kidney, brain, and pancreas [2, 6]. In contrast to the expression in tumor cells, the GnRH receptor is expressed only at low levels in cells of healthy organs, including the prostate, testes, and ovary, but is not present on most normal tissues [6–9]. This indicates a broad connection between cancerous transformation and GnRH receptor expression. Therefore, noninvasive imaging of the expression of the GnRH receptor would be of great help for the early detection of cancer, the evaluation of tumor progression, and monitoring of cancer treatment efficacy.

Advancement in imaging techniques such as positron-emission tomography (PET) can monitor in vivo molecular events noninvasively and provide functional information on diseases [10]. Over the past two decades, several studies reported the development of radiolabeled GnRH peptides for targeting GnRH receptors [11–18], but only a few have been successfully imaged by PET or SPECT [15–18]. In 2010, Jalilian et al. reported that [^111^In]-DTPA-buserelin was prepared for GnRH receptor studies but was only imaged and evaluated in normal rat with SPECT [15]. The next year, Guo et al. reported the design and synthesis of ^111^In-DOTAAhx-(d-Lys^6^-GnRH1) as a potential SPECT probe for breast cancer imaging [16]. In 2017, Xu et al. reported a similar SPECT probe ^111^In-DOTA-Aoc-d-Phe-(d-Lys^6^-GnRH) and imaged in DU145 human prostate cancer-xenografted nude mice [18]. GnRH peptide analogues labeled with positron-emitting radionuclides are of particular interest due to the high spatial resolution and sensitivity of PET [19]. Recently, Zoghi et al. developed [^68^Ga]-labeled DOTA-triptorelin as a possible PET radiotracer for GnRH receptor imaging [17]. The biodistribution of the tracer was determined in normal male rats and 4T1 tumor-bearing mice. However, neither PET imaging nor the uptake data of the 4T1 tumor were reported for the tracer.

In this study, we introduce the prosthetic group of 4-nitrophenyl-2-[^18^F]fluoropropionate ([^18^F]NFP) to the *ε*-amino group of the d-lysine side chain of d-Lys^6^-GnRH (pGlu^1^-His^2^-Trp^3^-Ser^4^-Tyr^5^-d-Lys^6^-Leu^7^-Arg^8^-Pro^9^-Gly^10^-NH_2_), which has demonstrated higher binding affinity and stability than native GnRH [20, 21]. The resulting probe, [^18^F]FP-d-Lys^6^-GnRH, was evaluated in a PC-3 human prostate cancer-xenografted model using micro-PET imaging. Extensive in vitro, ex vivo, and in vivo experiments were performed to evaluate its usefulness and pharmacokinetics as a PET tracer for noninvasive imaging of GnRH receptor expression.

## 2. Materials and Methods

### 2.1. General

All commercially available materials were used without further purification. The d-Lys^6^-GnRH [(Glp)-HWSY(D)KLRPG-NH_2_], [His^5^, d-Tyr^6^]GnRH [(Glp)-HWSH(D)YLRPG-NH_2_], and NOTA-P-d-Lys^6^-GnRH (*P* = PEG_3_) were custom manufactured from GL Biochem Ltd. (Shanghai, China). Sep-Pak light QMA and Plus C18 cartridges were obtained from Waters Corporation (Milford, MA, USA). High-performance liquid chromatography (HPLC) separation was performed on the PET-MF-2V-IT-I synthesizer module (PET Co. Ltd., Beijing, China) built-in HPLC system with a semipreparative reverse-phase C18 column (10 × 250 mm). Analytical HPLC was performed using a LC-20AD HPLC system (Shimadzu, Japan) equipped with a ZORBAX Eclipse XDB-C18 analytic column (4.6 × 150 mm, 5 *μ*m; Agilent Technologies, USA). Radioactivity was measured by a calibrated ion chamber (Capintec CRC-15R, Capintec, Inc. NJ, USA) or a gamma counter (*γ*-counter) (CAPRAC-R, Capintec, Inc. NJ, USA). High-resolution mass spectra were recorded with a Thermo Fisher Scientific Orbitrap Fusion mass spectrometer. No-carrier-added ^18^F-fluoride was obtained by reacting ^18^O(*p*, *n*)^18^F in a GE PET trace cyclotron with irradiating enriched [^18^O]water (98%). Statistical analysis was performed using Microsoft Office Excel. Continuous variables were analyzed using the Student's *t*-test with a significance level of *P* value less than 0.05.

### 2.2. Synthesis of NFP

The synthesis of NFP was performed as described previously [22]. In brief, to a solution of 2-fluoropropionic acid (75 mg, 0.82 mmol) in dry DMSO (1 mL) diisopropylethylamine (DIPEA, 100 *μ*L) and *Bis*(4-nitrophenyl) carbonate (259 mg, 0.82 mmol) were added at room temperature. The reaction mixture was stirred at 60°C for 3 h and was then cooled to room temperature and quenched with 5% acetic acid solution (1 mL). The product was purified by flash chromatography on silica gel (EtOAc: hexanes = 1 : 1) to yield the title compound as a red solid (61 mg, 0.54 mmol, 66% yield). NMR spectroscopy: ^1^H NMR (400 MHz, CDCl_3_) *δ* 8.30 (d, *J* = 8.8 Hz, 2H), 7.36 (d, *J* = 8.8 Hz, 2H), 5.30 (dd, *J* = 48.1, 6.9 Hz, 1H), and 1.77 (dd, *J* = 23.4, 6.9 Hz, 3H). ^13^C NMR (100 MHz, (CD_3_)_2_SO, 25°C, *δ*): 168.1 (d, *J* = 24 Hz), 154.6, 145.8, 125.3, 122.3, 85.4 (d, *J* = 183 Hz), and 18.3 (d, *J* = 22 Hz).

### 2.3. Synthesis of FP-d-Lys^6^-GnRH

NFP-labeled d-Lys^6^-GnRH was prepared according to a published procedure with modifications [22]. In brief, to a solution of d-Lys^6^-GnRH (5 mg, 4.0 *μ*mol in 0.2 mL of DMSO and 40 *μ*L of DIPEA), NFP (0.85 mg, 4.0 *μ*mol in 0.1 mL of DMSO) was slowly added at room temperature. The reaction mixture was stirred at room temperature for 3 h. The desired product was purified by semipreparative HPLC and lyophilized to yield FP-d-Lys^6^-GnRH as a white powder (3.2 mg, 2.4 *μ*mol, 60% yield). Analytical HPLC (*R*_t_ = 13.9 min) and HRMS (m/z: calculated for C_62_H_87_FN_18_O_14_ ([M + 2H]^2+^), 664.3383; found, 664.3389) analyses confirmed the product identification.

### 2.4. Radiosynthesis of [^131^I](His^5^, d-Tyr^6^)GnRH

[^131^I](His^5^, d-Tyr^6^)GnRH was prepared according to a similar published procedure [11, 14]. (His^5^, d-Tyr^6^)GnRH (100 *μ*g) in 100 *μ*L of Tris-HCl (25 mM, 0.4 M NaCl, PH 7.5) was reacted with Na^131^I (37 MBq; HTA CO. LTD. China), NaOH (10 *μ*L, 0.05 M), and chloramine-T (50 *μ*L, 1 mg/mL) for 30 min at room temperature. The reaction was terminated by the addition of Na_2_S_2_O_5_ solution (10 *μ*L, 1 mg/mL). Iodinated peptide was purified by HPLC with a gradient of 95% solvent A and 5% solvent B (0–2 min) ramped to 50% solvent A and 50% solvent B at 50 min.

### 2.5. In Vitro Receptor Binding Assay

GnRH membrane preparations were obtained from Millipore ChemiScreen (HTS027M). The GnRH receptor binding affinities (IC_50_ values) of FP-d-Lys^6^-GnRH, d-Lys^6^-GnRH, and NOTA-P-d-Lys^6^-GnRH were determined by in vitro competitive binding assay according to the manufacturer's protocol. In brief, 5 *μ*L of Millipore ChemiScreen human GnRH membrane preparations was incubated at room temperature for 1.5 h with approximately 20,000 counts per minute (cpm) of [^131^I](His^5^, d-Tyr^6^)GnRH in the presence of 10^−11^ to 10^−5^ M of each peptide in 95 *μ*L of binding buffer (50 mM HEPES, 5 mM MgCl_2_, 1 mM CaCl_2_, 0.2% BSA, buffer to pH 7.4 using 1 M NaOH). After the incubation, 600 *μ*L of ice-cold washing buffer (50 mM HEPES, 500 mM NaCl, 0.1% BSA, buffer to pH 7.4 using 1 M NaOH) was added to each mixture. Each resulting mixture was filtered through a GF/C filter (Whatman, Clifton, NJ) presoaked in 0.33% polyethylenimine. Each filter was rinsed with 1 mL of ice-cold wash buffer six times. The activities on the filters were measured in a gamma counter (*γ*-counter).

### 2.6. Radiosynthesis of [^18^F]FP-d-Lys^6^-GnRH

[^18^F]NFP was prepared and used for peptide radiolabeling based on a previously reported procedure [23]. In brief, a solution of d-Lys^6^-GnRH (50 *μ*g) in DMSO (200 *μ*L) and DIPEA (40 *μ*L) was added to anhydrous [^18^F]NFP. The reaction mixture was stirred for 10 min at 40°C and was diluted with water (10 mL). The dilution was passed through a Plus C18 cartridge, and then the cartridge was washed with water (10 mL). The desired radiolabeled compound was eluted with 2 mL of ethanol. For in vitro and in vivo experiments, the solvent was evaporated in vacuo and [^18^F]FP-d-Lys^6^-GnRH was formulated with 0.9% saline.

### 2.7. Octanol/Water Partition Coefficient

Approximately 740 KBq of [^18^F]FP-d-Lys^6^-GnRH was added to a vial containing 5 mL each of *n*-octanol and phosphate-buffered saline (PBS) (pH = 7.4). The mixture was vigorously vortexed for 1 min and centrifuged for 4 min to ensure the complete separation of layers. After centrifugation, three hundred milliliters of each layer were measured using a *γ*-counter, and logP values were calculated (*n*=3).

### 2.8. In Vitro Stability

An aliquot of [^18^F]FP-d-Lys^6^-GnRH (2.22 MBq, 10 *μ*L) in normal saline was added to PBS (200 *μ*L) or bovine serum (200 *μ*L) and incubated at 37°C for 2 h. For the PBS study, an aliquot of the solution was directly injected into a radio-HPLC for analysis. The bovine serum sample was filtered through a 0.22-*μ*m Millipore filter and injected into a radio-HPLC column for analysis.

### 2.9. Metabolic Stability

The metabolic stability of [^18^F]FP-d-Lys^6^-GnRH was evaluated in tumor-bearing nude mice according to a published procedure [24] with some modifications. Approximately 10 MBq of [^18^F]FP-d-Lys^6^-GnRH was injected intravenously in a conscious nude mouse bearing a PC-3 tumor as the control group (*n*=2 per group). For the blocking experiment, the tumor-bearing mice were coinjected with 15 mg/kg mouse body weight of d-Lys^6^-GnRH and 10 MBq of [^18^F]FP-d-Lys^6^-GnRH (*n*=2 per group). The mice were sacrificed at 1 h after injection, blood was obtained retro-orbitally, and the tumors were removed. The blood was then centrifuged (12000 g, 10 min). Tumors were separately homogenized in 0.5 mL of ethanol and centrifuged (12000 g, 10 min). The supernatant was filtered through a 30 KD Millipore filter and analyzed by HPLC. HPLC samples were collected in 0.5 min fractions for 30 min. The samples were counted in a *γ*-counter for 20 s. The counts were decay corrected to the injection time and added together. The counts were as the plotted intensity (cpm) versus fractions to show the profile of the sample. The data were plotted to reconstruct the HPLC spectrum.

### 2.10. Cell Culture and Animal Models

PC-3 human prostate cancer, SKBR-3 breast tumor, and CHO-K1 cell lines were purchased from the Institute of Biochemistry and Cell Biology, Shanghai Institutes for Biological Sciences, Chinese Academy of Sciences (Shanghai, China). Tumor cells (2−5 × 10^6^) were injected subcutaneously and allowed to grow for 2 to 4 weeks. At the time of the experiments, the tumor reached 3−6 mm (diameter), and the mice were 6–8 weeks old and weighed 16–24 g. All studies were approved by the Nanfang Hospital Animal Ethics Committee at the Southern Medical University.

### 2.11. Cell Uptake

PC-3 and CHO-K1 cells were seeded into 12-well plates at a density of 5 × 10^5^ cells/well and incubated overnight. In the cell uptake experiment, PC-3 and CHO-K1 cells were washed thrice with PBS, and then [^18^F]FP-d-Lys^6^-GnRH or with 10 *μ*M d-Lys^6^-GnRH was added in quadruplicate (1.3 *μ*Ci/well). After incubation at 37°C for 30 min, cells were rinsed thrice with PBS and lysed with NaOH-SDS (sodium dodecyl sulfate) (0.2 M NaOH, 1% SDS). The cell lysate was collected and measured in a *γ*-counter.

### 2.12. Micro-PET Studies

Dynamic micro-PET studies with tumor-bearing mice were performed using the Inveon MicroPET/CT scanner. A total of 5.55–7.4°MBq of [^18^F]FP-d-Lys^6^-GnRH was injected intravenously into animals via the tail vein. For the blocking experiment, the tumor-bearing mice were coinjected with 15 mg/kg mouse body weight of d-Lys^6^-GnRH and [^18^F]FP-d-Lys^6^-GnRH (*n*=3 per group). Dynamic scans were conducted over a period of 2 h. PET images were reconstructed using a three-dimensional ordered-subset expectation maximum algorithm. For data analysis, the region of interests (ROIs) were manually drawn over the tumor and major organs on decay-corrected whole-body coronal images using vendor software.

### 2.13. In Vivo Biodistribution

The PC-3 xenograft-bearing nude mice (*n*=4 for each group) were injected with approximately 1.0°MBq of [^18^F]FP-d-Lys^6^-GnRH via the tail vein. Animals were sacrificed at 120 min postinjection. The tumor and the other tissue samples of interest were rapidly dissected and weighed. ^18^F radioactivity was counted with a *γ*-counter. The results were calculated as the percentage injected dose per gram tissue or organ (% ID/g) (*n*=4).

### 2.14. Immunohistochemistry

The immunohistochemistry staining was performed on PC-3 and SKBR-3 cancer-xenografted tumors to demonstrate the GnRH receptor expression. The immunoperoxidase staining of the xenografted tumor slices (4 *μ*m thickness) were performed according to the protocol for the envision horseradish peroxidase system (DAKO EnVision System K5007) following the manufacturer's protocol. The primary antibody used in this study was GnRH receptor (Abcam ab183079, 1 : 50). Negative (no primary antibody) controls were included in the staining experiment. The cytoplasmic staining of the GnRH receptor was recorded as negative, weak, moderate, and strong [25].

## 3. Results

### 3.1. Chemistry

Nonradioactive FP-d-Lys^6^-GnRH was used as a reference standard for radiosynthesis and for receptor binding assays. FP-d-Lys^6^-GnRH was prepared by direct conjugation of NFP to d-Lys^6^-GnRH peptide with a yield of 52%. The product was purified by semipreparative RP-HPLC to achieve greater than 95% purity, and the correct molecular masses were confirmed by using orbitrap fusion mass spectrometry.

### 3.2. In Vitro Receptor Binding Assay

**Figure 1:**
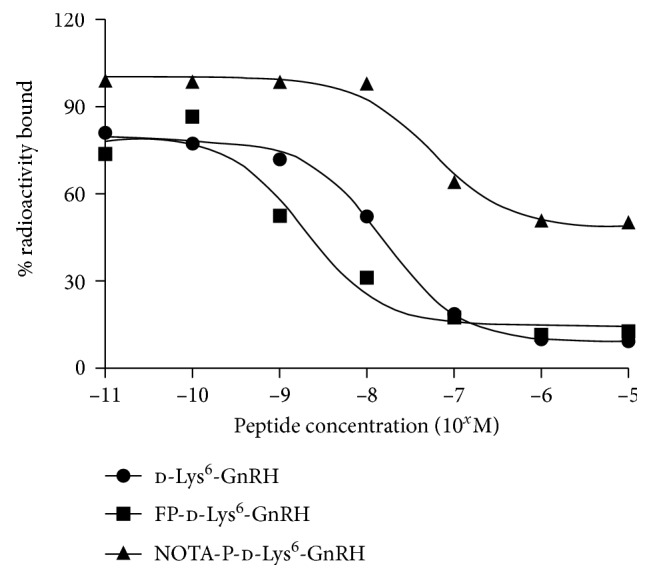
The competitive binding curves of d-Lys^6^-GnRH, FP-d-Lys^6^-GnRH, and NOTA-P-d-Lys^6^-GnRH. Data represent one of three separate experiments.

The GnRH receptor affinities for FP-d-Lys^6^-GnRH, d-Lys^6^-GnRH, and NOTA-P-d-Lys^6^-GnRH were determined using human GnRH membrane preparations and [^131^I](His^5^, d-Tyr^6^)GnRH as the radioligand. The IC_50_ values of FP-d-Lys^6^-GnRH, d-Lys^6^-GnRH, and NOTA-P-d-Lys^6^-GnRH were 2.0 nM, 15.8 nM, and 56.2 nM, respectively (*n*=3) (Figure 1). Since FP-d-Lys^6^-GnRH showed the strongest GnRH receptor binding affinity, we further evaluated [^18^F]FP-d-Lys^6^-GnRH.

### 3.3. Radiochemistry

**Figure 2:**
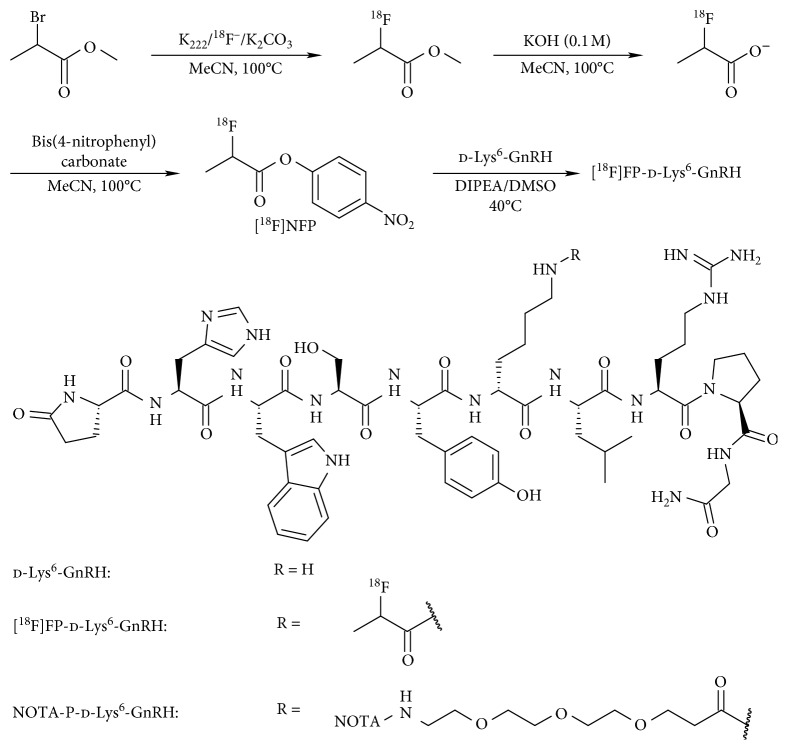
The multistep radiosynthesis of [^18^F]FP-d-Lys^6^-GnRH and the structures of GnRH analogues.

For the radiosynthesis of [^18^F]FP-d-Lys^6^-GnRH, we followed our previously published four-step two-pot methodology [23]. After ^18^F-fluorination of the bromo precursor and its hydrolysis with KOH, the 2-[^18^F]fluoropropanoate salt was activated with bis(4-nitrophenyl)carbonate to yield the [^18^F]NFP. Using 40 nmol of d-Lys^6^-GnRH was coupled to [^18^F]NFP at 40°C for 10 min (Figure 2). The product, [^18^F]FP-d-Lys^6^-GnRH, was isolated by solid-phase extraction without the need for HPLC separation with an overall yield of 8 ± 3% (decay corrected, referred to [^18^F]fluoride) and high specific activities of 20–100 GBq/*µ*mol (*n*=6).

### 3.4. Octanol/Water Partition Coefficient

The lipophilicity of [^18^F]FP-d-Lys^6^-GnRH was determined using the *n*-octanol/PBS distribution method. The logP_7.4_ value for the [^18^F]FP-d-Lys^6^-GnRH was -2.13 ± 0.04 (*n*=3).

### 3.5. In Vitro Stability and In Vivo Metabolism

**Figure 3:**
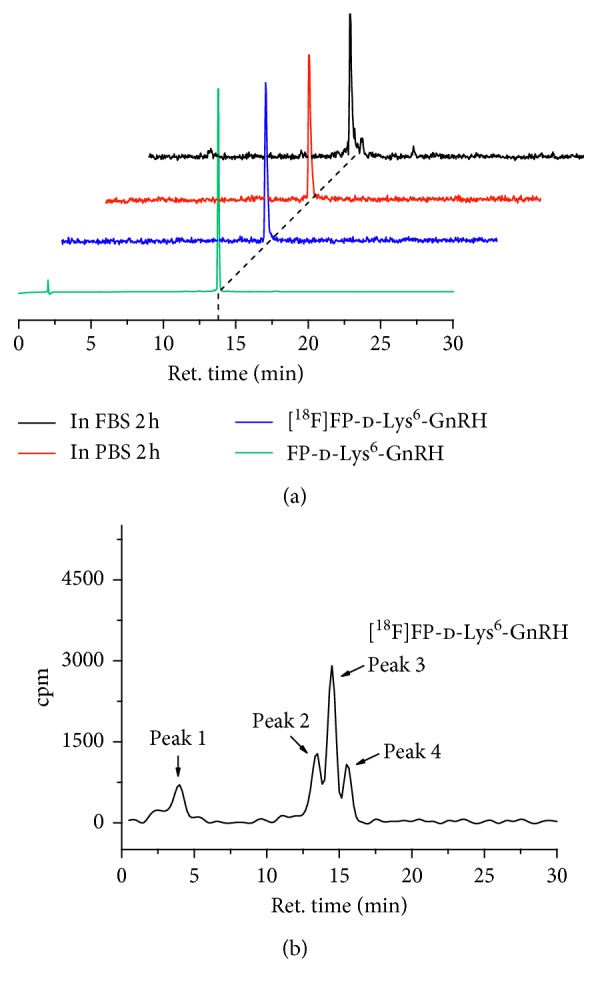
HPLC analysis the stability of [^18^F]FP-d-Lys^6^-GnRH: (a) [^18^F]FP-d-Lys^6^-GnRH incubation in PBS and bovine serum for 2 h compared to [^18^F]FP-d-Lys^6^-GnRH from the quality control and its reference standard FP-d-Lys^6^-GnRH; (b) representative HPLC profiles displaying unchanged [^18^F]FP-d-Lys^6^-GnRH (peak 3) and its metabolites (peak 1, peak 2, and peak 4) in PC-3 tumors at 1 h after intravenous injection in the blocking group. ret. = retention time in min.

To complete the determination of the in vitro characteristics of [^18^F]FP-d-Lys^6^-GnRH, the stability was determined by incubation of the radiotracer in PBS and bovine serum at 37°C for 2 h. [^18^F]FP-d-Lys^6^-GnRH showed high stability for up to 120 min, as determined by radio-HPLC analysis, and the unchanged tracer was greater than 95% (Figure 3(a)). Metabolites of [^18^F]FP-d-Lys^6^-GnRH in vivo after 1 h were analyzed in the PC-3 tumor-bearing mouse (Figure 3(b)). The results are summarized in Table 1. The extraction efficiency of tumor and blood was between 67% and 72% in both control and blocking groups (Table 1). In the control group, 23 ± 1.2% and 33 ± 0.10% of radioactivity were unchanged tracer in the supernatant of blood and tumor, respectively. Blockade of GnRH receptors with d-Lys^6^-GnRH resulted in an increment in unchanged tracer proportion by 41 ± 6.7% and 43 ± 2.2% in the supernatant of blood and tumor, respectively.

### 3.6. Cell Uptake

**Figure 4:**
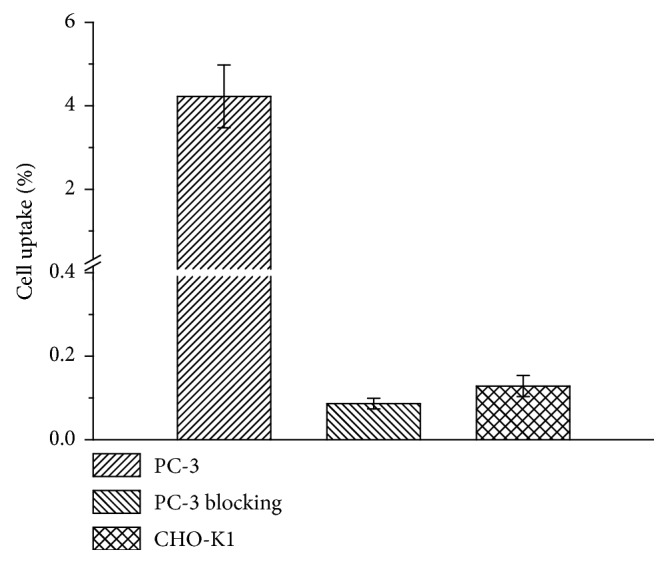
Cell uptake studies using PC-3 tumor cells (GnRH receptor-positive) and CHO-K1 cells (GnRH receptor-negative) (*n*=3, mean ± SD). Blocking studies with 10 *μ*M d-Lys^6^-GnRH confirmed the receptor-specific uptake.

The cell uptake of [^18^F]FP-d-Lys^6^-GnRH was evaluated in PC-3 human prostate GnRH receptor-positive cells and GnRH receptor-negative cells, CHO-K1. The results are shown in Figure 4. PC-3 cells express a high level of GnRH [26], and the uptake of [^18^F]FP-d-Lys^6^-GnRH is rapid and high, reaching approximately 4% within 30 min of incubation. By contrast, due to the relatively low GnRH receptor density of CHO-K1 cells [27], [^18^F]FP-d-Lys^6^-GnRH had relatively low cell uptake (<0.3%). Incubation of 10 *μ*M of d-Lys^6^-GnRH peptide blocked the PC-3 cells uptake (<0.3%), indicating that the binding of [^18^F]FP-d-Lys^6^-GnRH was GnRH receptor-specific.

### 3.7. Micro-PET Imaging Studies

**Figure 5:**
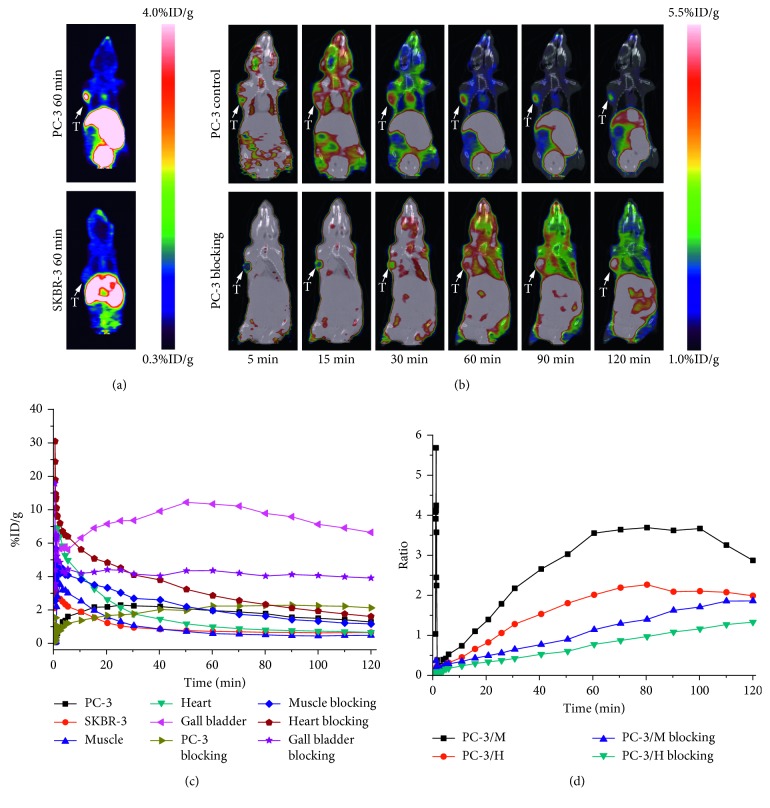
(a) Micro-PET images of [^18^F]FP-d-Lys^6^-GnRH in nude mice bearing PC-3 and SKBR-3 tumors at 60 min after intravenous injection. (b) MicroPET/CT images of [^18^F]FP-d-Lys^6^-GnRH in nude mice bearing the PC-3 tumor at 5, 15, 30, 60, 90, and 120 min after intravenous injection for control and blocking groups. Images are shown in coronal views. Tumors are indicated by arrowheads. (c) Time-activity curves illustrate [^18^F]FP-d-Lys^6^-GnRH dynamics in selected ROIs. (d) Comparison of the tumor to muscle and heart ratios of [^18^F]FP-d-Lys^6^-GnRH in nude mice bearing PC-3 tumors from 0 to 120 min after intravenous injection for control and blocking groups (*n*=3/group). T: tumor; M: muscle; H: heart.

Dynamic micro-PET studies for nude mice bearing PC-3 and SKBR-3 tumor xenografts were performed with [^18^F]FP-d-Lys^6^-GnRH (Figure 5). Animal PET images of summed 60 min coronal sections were selected for visualization (Figure 5(a)). As the images demonstrate, tumors could be visualized within each animal model. [^18^F]FP-d-Lys^6^-GnRH had higher tumor uptake in the PC-3 prostate tumor model than the SKBR-3 breast tumor model. Furthermore, blocking experiments were performed on PC-3 tumor-bearing mice. Representative coronal images of PC-3 tumor mice at 5, 15, 30, 60, 90, and 120 min after injection of [^18^F]FP-d-Lys^6^-GnRH in the presence of d-Lys^6^-GnRH (15 mg/kg) are illustrated in Figure 5(b). As the images showed, the tumor uptake in the control group was faster and more clearly visible compared with the blocking group. High gall bladder, kidney, and bladder uptake was observed in the animal model. To confirm this finding, a region-of-interest (ROI) analysis was performed on the reconstructed images to generate the time-activity curves for [^18^F]FP-d-Lys^6^-GnRH (Figure 5(c)). For the control group of the PC-3 tumor model, rapid tumor uptake was visualized in the first 15−20 min. Maximum tumor uptake was reached within the first 20 min after injection. Tumor uptake then slowly washed out throughout the 120 min scan time, suggesting that the radioactivity was taken up and trapped in the tumor tissue. In contrast, the presence of a blocking dose of d-Lys^6^-GnRH reduced the tumor uptake during the first 50 min. After that, the tumor uptake was higher than the control group and remained consistent throughout the 120 min scan time, with little to no washout observed. The absolute radioactivity accumulation in the muscle and heart of the blocking group was higher than that observed in the control group. The ratios of tumor-to-muscle and tumor-to-heart in the blocking group were lower than the control group throughout the 120 min scan time (Figure 5(d)). Tumor-to-muscle and tumor-to-heart ratios were 3.55 and 2.01, respectively, at 1 h i.v. for the control group. In comparison, the ratios for tumor-to-muscle and tumor-to-heart for the blocking group were 1.14 and 0.78, respectively.

### 3.8. Biodistribution Studies

**Figure 6:**
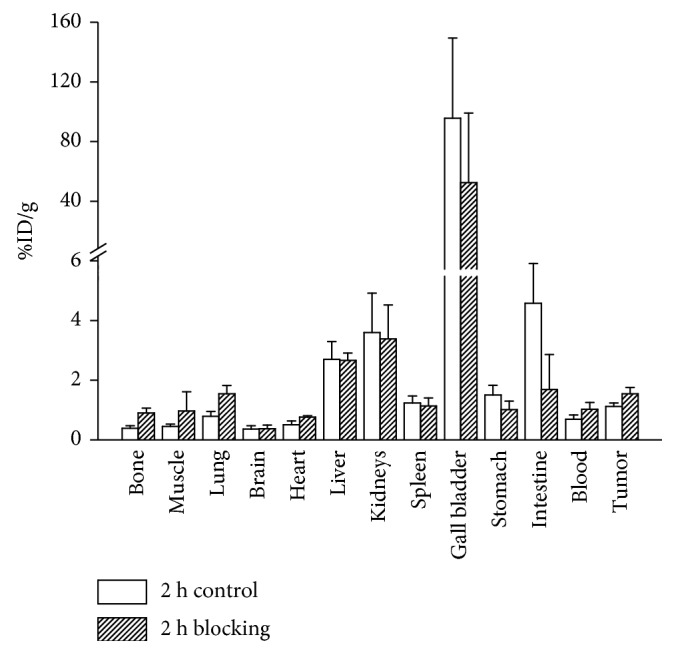
Biodistribution data of [^18^F]FP-d-Lys^6^-GnRH in nude mice bearing PC-3 tumors at 1 h postinjection. All data are expressed as the mean values ± SD (*n*=4).

To validate the micro-PET imaging experiments, biodistribution studies of [^18^F]FP-d-Lys^6^-GnRH were performed at 2 h after intravenous dosing in PC-3 xenograft models. The results are shown in Figure 6. No significant differences were observed compared to the micro-PET image quantification. Compared to the control group, [^18^F]FP-d-Lys^6^-GnRH in the blocking group resulted in higher uptake in the organs, except the gall bladder, kidney, intestine, stomach, and spleen. These results indicate that the tracer excretes through the gall bladder system and that the metabolic rate was slow in excess d-Lys^6^-GnRH. Although PC-3 tumor uptake in the blocking group was higher than in the control group, the ratio of tumor-to-muscle in the control group was higher than in the blocking group (2.5 vs 1.6, *p* < 0.05).

### 3.9. Immunohistochemistry

**Figure 7:**
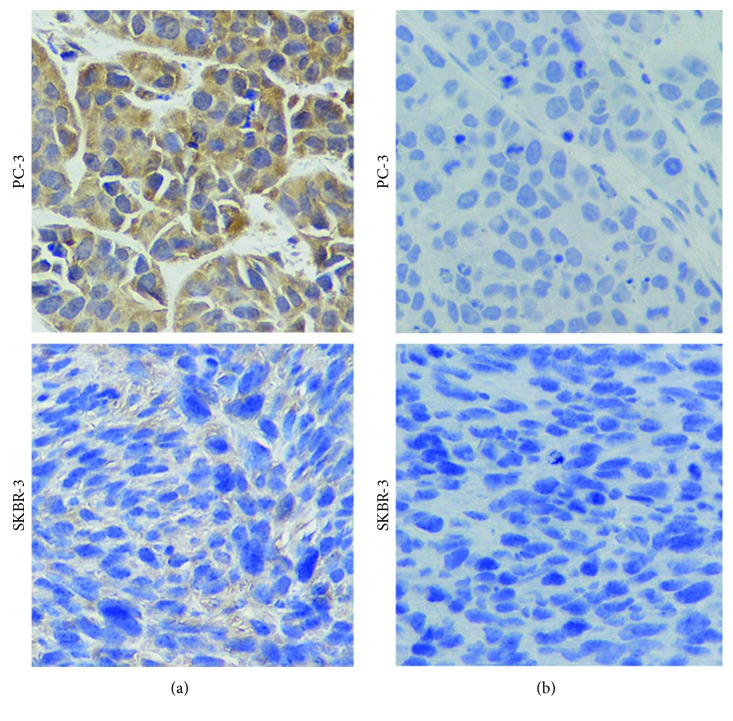
Immunohistochemical staining of GnRH receptor expression in PC-3 and SKBR-3 xenografted tumors: (a) the PC-3 xenografted tumor exhibited strong brown cytoplasmic staining and the SKBR-3 xenografted tumor exhibited weak brown cytoplasmic staining; (b) the PC-3 and SKBR-3 xenografted tumors were stained without primary goat antihuman GnRH antibody (×400).

The GnRH receptor expression in PC-3 prostate cancer and SKBR-3 breast tumor slices was confirmed by immunohistochemistry staining. The immunohistochemistry staining results are presented in Figure 7. The cytoplasmic staining of the GnRH receptor in PC-3 human prostate cancer was strong. In contrast, the cytoplasmic staining of GnRH receptor in SKBR-3 breast tumor was weak. Hence, we used the PC-3 and SKBR-3 tumor-bearing mice models to determine the tumor targeting and pharmacokinetic properties of [^18^F]FP-d-Lys^6^-GnRH.

## 4. Discussion

The expression of GnRH and its receptor in human malignant tumors of the urogenital tract is part of an autocrine system, which regulates cell proliferation [2]. GnRH receptor overexpression occurs in many malignancies and is a suitable target for cancer therapy [2, 3, 28–30]. As such, the in vivo imaging of GnRH receptor expression would serve as an imaging marker for tumor growth, providing critical diagnostic and prognostic information for the management of a patient with cancer.

Although a few radiolabeled peptides are being evaluated for tumor and GnRH receptor imaging [15–18], no successful PET tracer has been reported to our knowledge. The GnRH agonist d-Lys^6^-GnRH has served as a carrier to target chemotherapy agents or radionuclides to the GnRH receptor for cancer treatment or imaging [6, 9, 13, 14, 16, 26, 30–34]. In this study, we conjugated a small 2-fluoropropionate group to d-Lys^6^-GnRH (FP-d-Lys^6^-GnRH) and NOTA to the d-Lys^6^ via the PEG linker to d-Lys^6^-GnRH (NOTA-P-d-Lys^6^-GnRH). Receptor binding assays using membrane preparations and [^131^I](His^5^, d-Tyr^6^)GnRH as the radioligand showed that FP-d-Lys^6^-GnRH (IC_50_ = 2.0 nM) was approximately 8-fold higher in potency than the reference d-Lys^6^-GnRH (IC_50_ = 15.8 nM). The NOTA-P-d-Lys^6^-GnRH (IC_50_ = 56.2 nM) dramatically decreased the GnRH receptor binding affinity by approximately 4-fold. FP-d-Lys^6^-GnRH displayed 2.0 nM GnRH receptor binding affinity, warranting its further evaluation. We introduce ^18^F into d-Lys^6^-GnRH using the prosthetic group [^18^F]NFP, which as a small ^18^F-radiolabeling group for peptides has less influence on the characteristics of peptides [22]. Furthermore, some peptides labeled with [^18^F]NFP are successfully used in clinical trials in human subjects [35]. Moreover, in cell uptake studies using GnRH receptor-positive PC-3 cells and GnRH receptor-negative CHO-K1 cells, it could be demonstrated that [^18^F]FP-d-Lys^6^-GnRH was specifically binding to the GnRH receptor. The receptor-specific uptake is also confirmed by micro-PET imaging in PC-3 prostate and SKBR-3 tumor models. The GnRH receptor expression in the two model tumors was confirmed by immunohistochemistry staining.

[^18^F]FP-d-Lys^6^-GnRH was evaluated in the PC-3 prostate xenograft model and showed tumor uptake and primarily gall bladder system excretion routes. Liver uptake was significant and followed by biliary tract excretion, which was observed after 2 h, consistent with other radiolabeled peptides [15]. The radioactivity has moderate accumulation in the kidney and intestine, possibly because the hydrophilic metabolites of the [^18^F]FP-d-Lys^6^-GnRH were excreted through the kidneys. In the control group, [^18^F]FP-d-Lys^6^-GnRH exhibited rapid tumor uptake and reached a maximum at 20 min i.v. and then was slowly washed out. However, [^18^F]FP-d-Lys^6^-GnRH exhibited maximum tumor uptake at 60 min i.v., and sustained tumor accumulation was observed in the blocking group. Furthermore, muscle and heart uptake in the blocking group was higher than in the control group throughout the 120 min scan time, which was consistent with the literature reported for [^125^I]triptorelin [14]. In order to find the reason, the metabolic stability studies of [^18^F]FP-d-Lys^6^-GnRH were performed in the PC-3 tumor-bearing mouse. The proportion of radioactivity in the supernatant from blood and tumors was not significantly different in control and blocking groups. For the proportions of the unchanged tracer in blood and tumors, the blocking group were higher than in the control group. The results showed that excretion of the tracer was slow in the excess of d-Lys^6^-GnRH.

Through the in vivo and in vitro studies of [^18^F]FP-d-Lys^6^-GnRH developed in this study, we believe that ^18^F-labeled GnRH peptide is a potential tracer for GnRH expression. However, high radioactivity accumulation in the gall bladder and abdomen will limit its application in detecting lesions that are in the gall bladder and around the urinary bladder. Further optimization of the scaffold to reduce or eliminate radioactivity uptake in the abdomen is underway in our laboratories.

## 5. Conclusions

We successfully radiolabeled d-Lys^6^-GnRH with the small prosthetic group [^18^F]NFP. In vitro assays, in vivo imaging, and biodistribution studies showed that [^18^F]FP-d-Lys^6^-GnRH exhibited high affinity for the GnRH receptor, predominant gall bladder system elimination, and specific tumor uptake in tumor xenografts. As a result, [^18^F]-labeled GnRH peptide exhibits potential for PET imaging of GnRH receptor expression.

## Figures and Tables

**Table 1:** Proportions of supernatant and unchanged [^18^F]FP-d-Lys^6^-GnRH in nude mice bearing PC-3 tumor at 1 h after intravenous injection for control and blocking groups.

	Blood	Tumor	15 mg/kg	
			Blood	Tumor
Supernatant (%)	72 ± 8.2	67 ± 15	71 ± 8.8	72 ± 18
Unchanged tracer (%)	23 ± 1.2	33 ± 0.10	41 ± 6.7	43 ± 2.2

## Data Availability

All data included in this study are available upon request from the first author or corresponding author.

## Supplementary Materials

Supplementary Materials Some original data of NMR, MS, and HPLC for the PET tracer of [^18^F]FP-d-Lys^6^-GnRH are provided in the supplementary information file. Figure S1: ^1^H and ^13^C-NMR spectra of 4-nitrophenyl-2-fluoropropionate (NFP). Figure S2: mass spectra of FP-d-Lys^6^-GnRH. Figure S3: semipreparative radio-high-performance liquid chromatography (radio-HPLC) of [^18^F]NFP. Figure S4: radio-HPLC of [^18^F]FP-d-Lys^6^ -GnRH for in vitro stability.
