# Supplementary material for: Synthesis and Evaluation of 18F-Labeled Peptide for Gonadotropin-Releasing Hormone Receptor Imaging
Source: Contrast Media Mol Imaging. 2019 Mar 7;2019:5635269. doi: 10.1155/2019/5635269 (PMC6431521; doi:10.1155/2019/5635269)

Supplementary Information

1.1 General

HPLC separation was performed on the PET-MF-2 V-IT-I synthesizer module (PET Co. Ltd., Beijing, China) built-in HPLC system with an S1021 pump (SYKAM Chromatography Vertriebs GmbH, Bayern, Germany), Well-ChromK-2001 UV detector (KNAUER Wissenschaftliche Gerate GmbH, Berlin, Germany), radioisotope detectors, and an Agilent ZORBAX SB-C18 9.4×250mm 5μ semi-preparative column. The mobile phase was 60% solvent A (0.1% TFA in water): 40% solvent B (0.1% TFA in MeCN) with a flow rate of 4 mL/min. Analytical HPLC was performed using a LC-20AD HPLC system (Shimadzu, Japan) equipped with a ZORBAX Eclipse XDB-C18 analytic column (4.6 × 150 mm, 5 μm; Agilent Technologies, USA) using a flow rate of 1 mL/min. The gradient program started from 95% solvent A (0.1% TFA in water) and 5% solvent B (0.1% TFA in MeCN) (0–2 min) and ramped to 20% solvent A and 80% solvent B at 25 min. The elution profile was detected with an ultraviolet detector (Agilent interface 35900E, Agilent Technologies, USA) at 210 nm and a B-FC-3200 high energy PMT Detector (Bioscan Inc, Washington DC, USA). NMR spectra were recorded on a Bruker 400 Nanobay spectrometer operating at 400 MHz and100 MHz for ^{1}H and ^{13}C acquisitions, respectively. Chemical shifts were referenced to the residual proton solvent peaks (^{1}H: $CDCl_3$, δ 7.26), solvent ^{13}C signals ($CDCl_3$, δ 77.16). High-resolution mass spectra were recorded with a ThermoFisher Scientific Orbitrap Fusion mass spectrometer.

1.2 Spectroscopic Data

Figure S1. ^{1}**H and** ^{13}**C-NMR spectra of 4-nitrophenyl-2-fluoropropionate (NFP)**

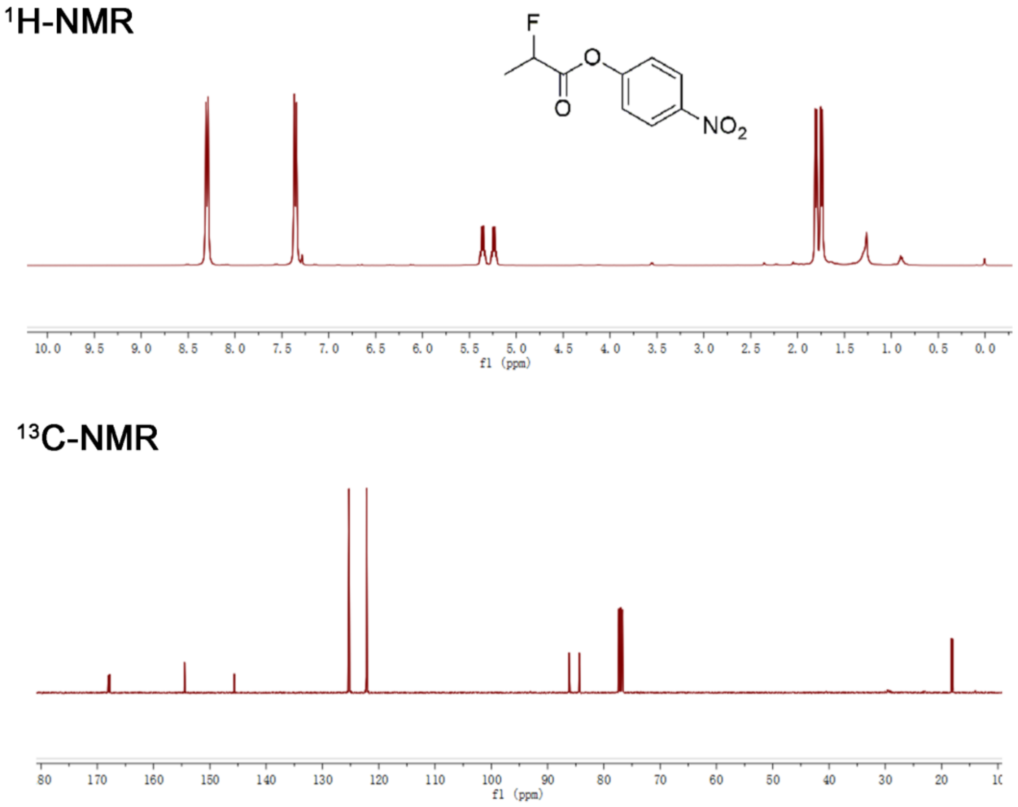

Figure S2. Mass spectra of FP-D-Lys^{6}**-GnRH**

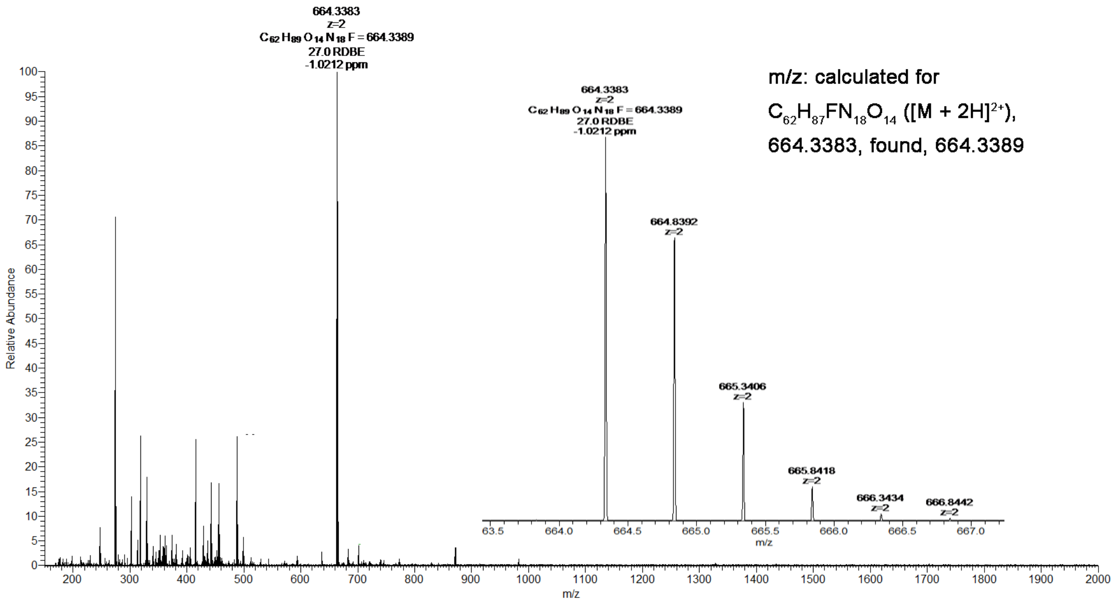

1.3 Chromatography Data

Figure S3. Semi-preparative radio-high-performance liquid chromatography (Radio-HPLC) of [^{18}F]NFP

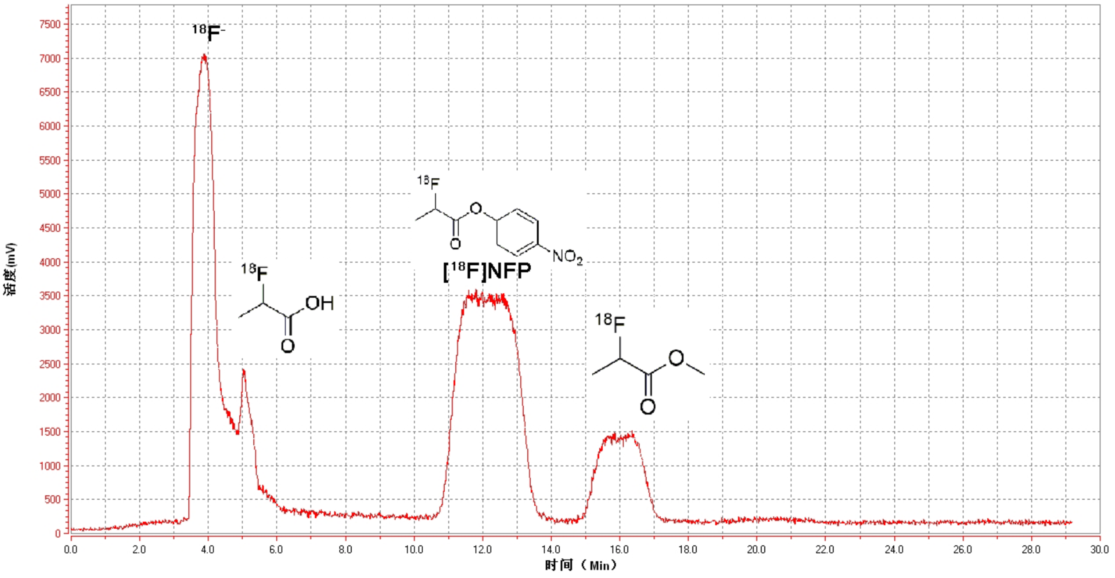

Figure S4. Radio-HPLC of [^{18}F]FP-D-Lys^{6}-GnRH for vitro stability

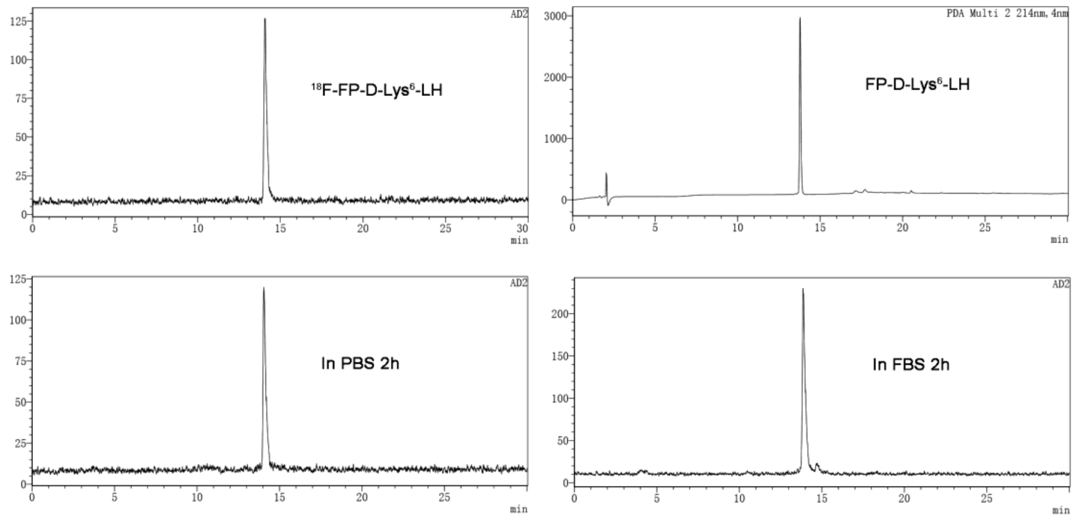

Supplement: Supplementary Materials — Some original data of NMR, MS, and HPLC for the PET tracer of [18F]FP-d-Lys6-GnRH are provided in the supplementary information file. Figure S1: 1H and 13C-NMR spectra of 4-nitrophenyl-2-fluoropropionate (NFP). Figure S2: mass spectra of FP-d-Lys6-GnRH. Figure S3: semipreparative radio-high-performance liquid chromatography (radio-HPLC) of [18F]NFP. Figure S4: radio-HPLC of [18F]FP-d-Lys6 -GnRH for in vitro stability. [file 5635269.f1.pdf]
